# The larvicidal effects of black pepper (*Piper nigrum* L.) and piperine against insecticide resistant and susceptible strains of *Anopheles* malaria vector mosquitoes

**DOI:** 10.1186/s13071-016-1521-6

**Published:** 2016-04-26

**Authors:** Michael Samuel, Shüné V. Oliver, Maureen Coetzee, Basil D. Brooke

**Affiliations:** Wits Research Institute for Malaria, School of Pathology, Faculty of Health Sciences, University of the Witwatersrand, Johannesburg, South Africa; Centre for Opportunistic, Tropical & Hospital Infections, National Institute for Communicable Diseases, Johannesburg, South Africa

**Keywords:** *An. arabiensis*, *An. coluzzii*, *An. funestus*, *An. gambiae*, *An. quadriannulatus*, Larvicides, Vector control

## Abstract

**Background:**

Insecticide resistance carries the potential to undermine the efficacy of insecticide based malaria vector control strategies. Therefore, there is an urgent need for new insecticidal compounds. Black pepper (dried fruit from the vine, *Piper nigrum*), used as a food additive and spice, and its principal alkaloid piperine, have previously been shown to have larvicidal properties. The aim of this study was to investigate the larvicidal effects of ground black pepper and piperine against third and fourth instar *Anopheles* larvae drawn from several laboratory-reared insecticide resistant and susceptible strains of *Anopheles arabiensis*, *An. coluzzii*, *An. gambiae*, *An. quadriannulatus* and *An. funestus*.

**Methods:**

Larvae were fed with mixtures of standard larval food and either ground black pepper or piperine in different proportions. Mortality was recorded 24 h after black pepper and 48 h after piperine were applied to the larval bowls.

**Results:**

Black pepper and piperine mixtures caused high mortality in the *An. gambiae* complex strains, with black pepper proving significantly more toxic than piperine. The *An. funestus* strains were substantially less sensitive to black pepper and piperine which may reflect a marked difference in the feeding habits of this species compared to that of the Gambiae complex or a difference in food metabolism as a consequence of differences in breeding habitat between species.

**Conclusions:**

Insecticide resistant and susceptible strains by species proved equally susceptible to black pepper and piperine. It is concluded that black pepper shows potential as a larvicide for the control of certain malaria vector species.

## Background

Malaria is responsible for high levels of morbidity and mortality globally, with particular severity in sub-Saharan Africa [1]. This region is home to several of the most efficient malaria vectors, the principal species being *Anopheles gambiae*, *An. coluzzii* (formerly *An. gambiae* M form [2]), *An. arabiensis* and *An. funestus*. The former three species are members of the *Anopheles gambiae* species complex and the latter is the nominal member and only major vector of the *Anopheles funestus* species group [3, 4].

Insecticide based vector control strategies primarily directed against adult mosquitoes have significantly decreased malaria incidence over the past decade, with indoor residual spraying of insecticides (IRS) and distribution of long-lasting insecticide-treated bednets (LLINs) being pivotal [1]. Additionally, and especially in light of the increasing incidence of insecticide resistance in target vector populations [5], larval source management (LSM) is playing an increasingly important role in vector control [6].

Larval source management as a vector control technique has dramatically decreased since the shift toward the use of synthetic mosquito adulticides, especially DDT and later pyrethroids – this despite distinct successes achieved by larviciding in the early-to-mid 1900’s [7]. LSM is currently only recognized as supplementary to the core interventions of IRS and LLINs by the World Health Organization (WHO), being employed in only 38 of the 97 countries with ongoing malaria transmission [1]. Nevertheless, the WHO encourages the use of LSM where conditions are appropriate and it is feasible to do so [8] and recent evidence, though sparse, does suggest that larviciding may have a reinvigorated, valuable role to play in integrated vector management [7, 9, 10]. It is for these reasons that investigations into potential options for use in larviciding are ongoing.

A tendency towards naturally occurring insecticides, particularly those of botanical origin, has gained increasing interest in recent years. Plants have co-evolved with insects over many centuries, as have their defence mechanisms in response to insect predation. Plant allelochemicals can be very advantageous as insecticidal actives compared to their synthetic counterparts as they are biodegradable and in many instances show reduced or no adverse effects on non-target organisms [11].

*Piper nigrum* L. (the fruit of which are used to produce white and black pepper) has a plethora of traditional and modern day applications ranging from a US FDA recognized food additive to potential as an insecticide [12]. While assessing spreading agents for *Metarhizium anisopliae* and *Beauveria bassiana* entomopathogenic fungal spores, Bukhari et al. [13] observed 100 % mortality of *Anopheles* larvae exposed to white pepper, even in the absence of the fungal spores. This has prompted an interest in the use of pepper as a possible larvicide for use in malaria vector control. In addition, piperine, which is the principle alkaloid responsible for the pungency of pepper, has demonstrated toxicity against fourth-instar larvae of the dengue and yellow fever vector *Aedes aegypti* [14, 15].

The aim of this study was to investigate the toxicity of *P. nigrum* (black pepper) and piperine when administered as a food source to larvae of several insecticide resistant and susceptible strains of *Anopheles* species including *An. arabiensis*, *An. coluzzii*, *An. gambiae, An. quadriannulatus* and *An. funestus*.

## Methods

### Mosquito strains

All of the mosquito strains used in this study are housed in the Botha De Meillon Insectary (BMDI) at the National Institute for Communicable Diseases (NICD) in Johannesburg. All larvae were reared and bioassays conducted under the standard insectary conditions [16]. Table 1 gives detailed information on each of the strains used.

**Table 1:** *Anopheles* species by laboratory strain and insecticide susceptibility profile fed on powdered formulations of either *Piper nigrum* (black pepper) or piperine at the larval stage

Species	Strain (Country of origin)	Resistance profile/additional information	Resistance mechanisms
*Anopheles arabiensis*	KGB (Zimbabwe)	Insecticide susceptible	N/A
	SENN (Sudan)	Mostly susceptible. Low-level resistance to permethrin	N/A [27]
	SENN-DDT (Sudan)	Selected for resistance to DDT from SENN base colony. Also resistant to permethrin, deltamethrin and malathion [27, 28]	Elevated cytochrome P450, glutathione S-transferase (GST) and general esterase activity [27, 28]
*Anopheles coluzzii*	SILC^a^ (Sierra Leone)	Resistant to pyrethroids and DDT	^a^No data
	SUA (Liberia)	Insecticide susceptible	N/A
*Anopheles gambiae*	GAH (Ghana)	Resistant to pyrethroids, DDT, carbamates and organophosphates [29]	Monooxygenase and esterase mediated detoxification coupled with the L1014F *kdr* mutation are implicated in pyrethroid resistance. An assortment of L1014F *kdr* is also implacted in DDT resistance. Mutations of the Alanine296-glycine (*Rdl*) GABA receptor and acetylcholinesterase receptor (*ace-1* ^*R*^) are associated with dieldrin and bediocarb resistance respectively [29].
	TONGS^a^ (Cote d’Ivoire)	Resistant to pyrethroids, DDT, carbamates and organophosphates	^a^No data
*Anopheles quadriannulatus*	SANGWE (Zimbabwe)	Insecticide susceptible. *Anopheles quadriannulatus* is zoophilic and not considered to be a vector species.	N/A
*Anopheles funestus*	FANG (Angola)	Insecticide susceptible	N/A
	FUMOZ-R (Mozambique)	Resistant to pyrethroids and carbamates [30]	Overexpression of the cytochrome P450 CYP6P9 [30]. Thickened cuticles also contribute to adult insecticide-resistance [31].

^a^SILC was used for black pepper bioassays but not for piperine bioassays as the insecticide resistance levels in the strain had significantly dropped during the period between the conducting of the two bioassays and it would not have been an accurate proxy by which to compare insecticide susceptible and resistant strains. SILC was replaced with the *An. gambiae* strain TONGS for the piperine bioassays

### Technical materials

Commercial ground black pepper was further homogenized in a Qiagen Tissue Lyser II, for 5 min, until a fine powdery consistency was achieved. Analytical grade piperine in powder form (expiry date: December 2016) was purchased from Sigma Aldrich (Saint Louis, MO). The technical materials were each proportionately mixed with standard larval food [16] to obtain treatment mixtures ranging from 0 % pepper/piperine (control) to 100 % pepper/piperine (Table 2).

**Table 2:** Proportions of black pepper (*Piper nigrum*) or piperine to standard larval food used as treatment dosages for *Anopheles* larval toxicity bioassays

Black pepper/piperine (mg)	Standard larval food (mg)	Dosage of black pepper/piperine (%)
0	50	0
5	45	10
10	40	20
20	30	40
40	10	80
50	0	100

### Larval toxicity bioassays

Fifty third to fourth instar larvae from each *Anopheles* strain were gently introduced into plastic containers containing 500 ml distilled water (surface area = 150 mm × 215 mm). Fifty milligram (1 mg treatment mixture/larva) of each treatment mixture, in addition to a control composed entirely of standard larval food, was tapped gently into each container to allow for an even spread over the surface of the water. Mortality was recorded 24 h after the application of the black pepper mixtures and 48 h after the piperine mixtures (little or no mortality was observed following 24 h larval exposure to piperine). At the end of the period, larvae were gently prodded and non-responsive larvae were recorded as dead [17]. Bioassays for each treatment mixture were replicated three times per strain***.***

### Statistical analysis

Mortality was corrected using Abbott’s formula in replicates where control mortality exceeded 10 %. One-way ANOVA and Tukey HSD *post-hoc* tests were used to determine (i) if the mortality in treated bioassays significantly differed from that of the controls and at which doses in particular; (ii) if there were significant differences in response between insecticide susceptible and resistant strains by species where pertinent; and (iii) if there were significant differences in response between species of the Gambiae complex and Funestus group. For the latter, analysis excluded control mortalities from the data. A Student’s *t*-test was used to determine if there were significant differences in response between intoxication with black pepper and piperine. All statistics were conducted in IBM SPSS Statistics v22 with significance set at 95 % confidence.

## Results

For this study “dose”, which is represented by a percentage, refers to the proportion (Table 2) of black pepper or piperine constituting 50 mg of treatment mixture administered to larvae.

### Black pepper

**Fig. 1:**
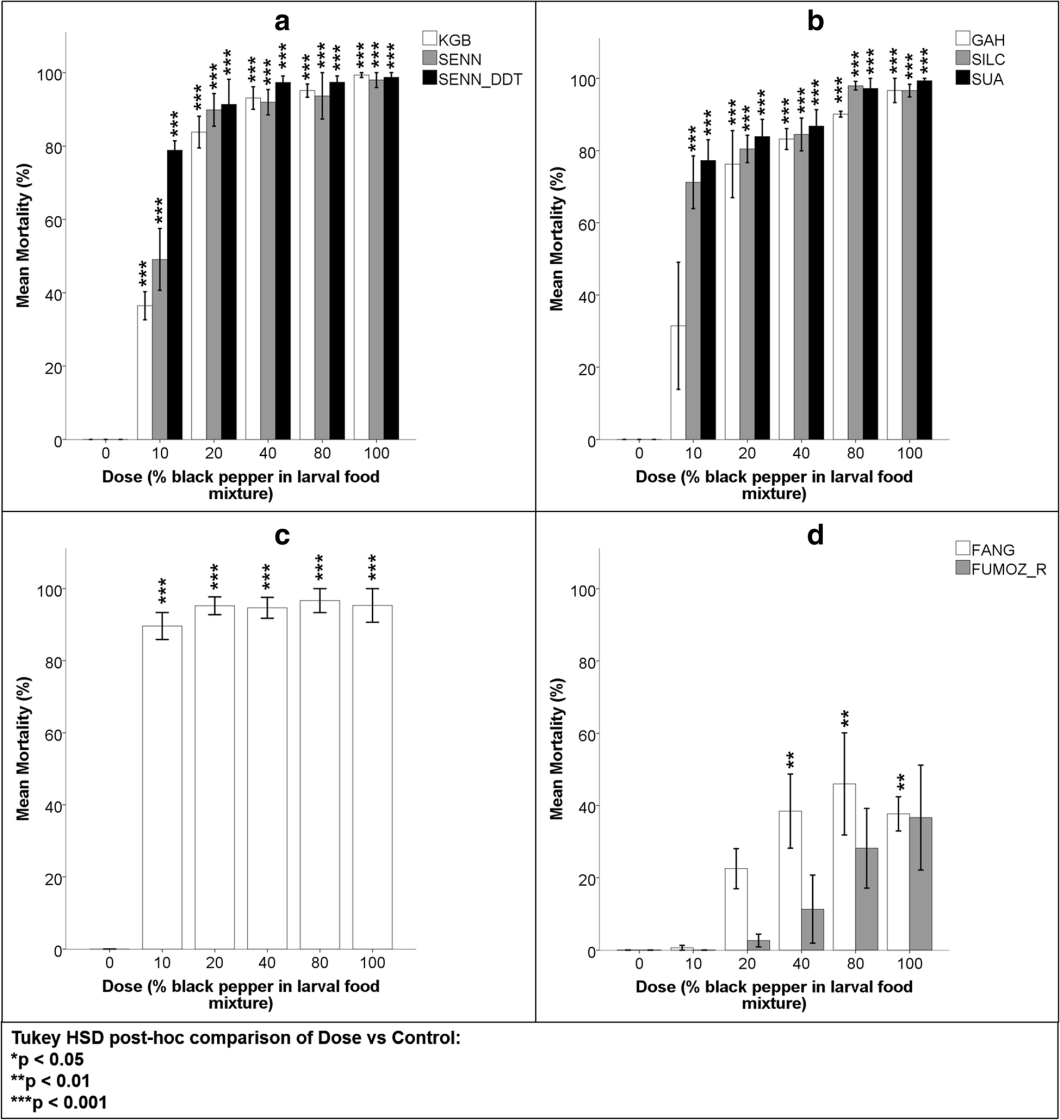
Mean mortalities of laboratory-reared *Anopheles* larvae 24 h after being fed powdered black pepper (*Piper nigrum*) at various concentrations. The dose value represents the percentage of black pepper in a 50 mg treatment mixture. 0 % represents the control group which was fed standard larval food only and 100 % represents a treatment comprised of black pepper only. A One-way ANOVA and Tukey HSD *post-hoc* comparisons were used for each strain to determine significant differences in mortality at each dose compared to that of the relevant control at 95 % confidence. **a** *Anopheles arabiensis* strains. **b** *Anopheles gambiae* (GAH) and *An. coluzzii* (SILC & SUA) strains. **c** *Anopheles quadriannulatus.* **d** *Anopheles funestus* strains

**Fig. 2:**
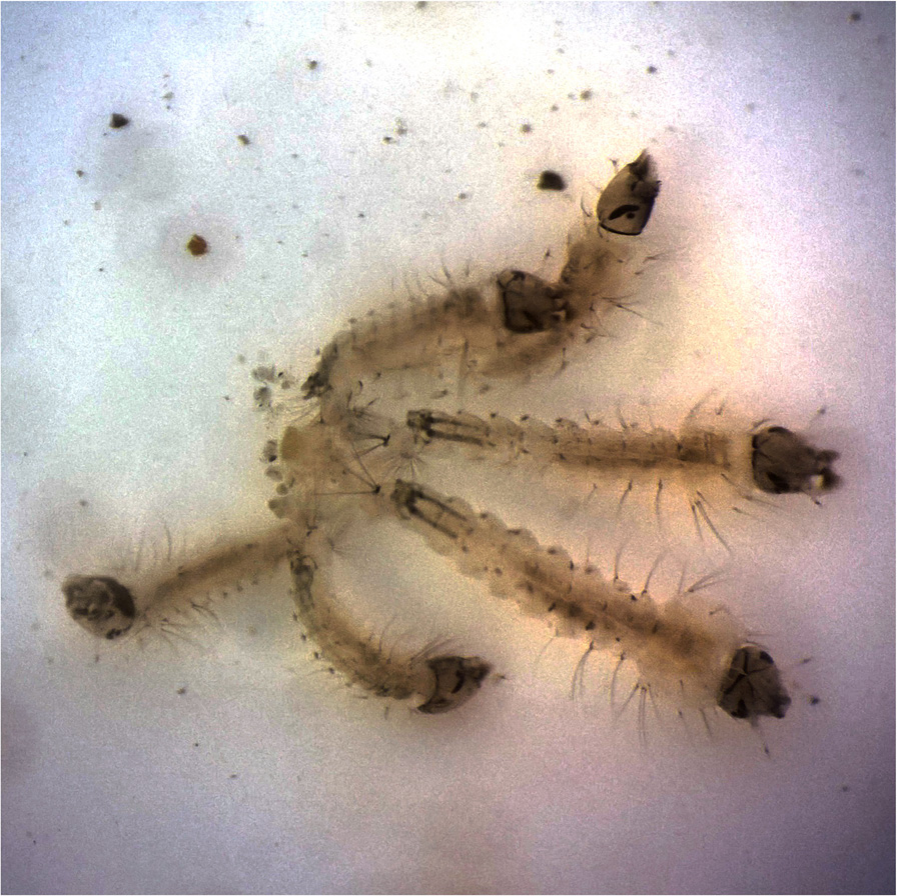
Clustering of dead *Anopheles quadriannulatus* larvae 24 h after being fed a powdered black pepper treatment mixture. Similar clustering was observed among all strains of all *An. gambiae* complex species tested

Black pepper, when applied to the water, tended to spread over the surface quite evenly and effectively. Mortality was induced in all species and strains by all treatments containing pepper (Fig. 1a–d). In general, mortality increased with increasing proportions of pepper in the treatment mixture. Furthermore, the presence of pepper (even in low quantities) consistently induced significantly higher mortalities in the *An. gambiae* complex larvae than in *An. funestus* (Table 3). Dead *An. gambiae* complex larvae tended to cluster, pivoting around their tracheal gills (Fig. 2).

**Table 3:** Tukey HSD *post-hoc* analysis of *Anopheles* larval mean mortalities 24 h post-exposure to black pepper (*P. nigrum*)

	Strain	Mean mortality	Standard deviation
1	KGB	81.59^8–9***^	24.35
2	SENN	84.54^8–9***^	20.17
3	SENN-DDT	92.75^8–9***^	9.170
4	GAH	75.55^8–9***^	27.38
5	SILC	86.18^8–9***^	12.20
6	SUA	88.93^8–9***^	10.47
7	SANGWE	94.30^8–9***^	5.723
8	FANG	29.07^1–7***^	20.78
9	FUMOZ-R	15.77^1–7***^	20.07

A one-way ANOVA (*P* < 0.01; *F* = 38.11 at *df* = 134), supplemented with a Tukey HSD *post-hoc* test, was used to determine significant differences in the overall mean mortalities induced between *Anopheles* strains indicated by superscript numbering at 95, 99 and 99.9 % confidence

***P < 0.001

#### *Anopheles arabiensis*

The treatment dose of 100 % black pepper achieved upwards of 98 % mortality in all three of the *An. arabiensis* strains and all of the treatment doses induced significantly higher mortality than the control (0 % black pepper) for KGB (ANOVA: *P* < 0.01; *F* = 211.51 at *df* = 17), SENN (ANOVA: *P* < 0.01; *F* = 62.05 at *df* = 17) and SENN-DDT (ANOVA: *P* < 0.01; *F* = 148.30 at *df* = 17) (Fig. 1a). There were no significant differences in mean mortality between SENN-DDT and either KGB or SENN (Table 3).

#### *Anopheles gambiae* and *An. coluzzii*

With the exception of GAH exposed to 10 % black pepper, all of the treatment mixtures achieved significantly higher mortality against larvae of both GAH (*An. gambiae*) (ANOVA: *P* < 0.01; *F* = 21.31 at *df* = 17) and the *An. coluzzii* strains SILC (ANOVA: *P* < 0.01; *F* = 86.25 at *df* = 17) and SUA (ANOVA: *P* < 0.01; *F* = 98.61 at *df * = 17) (Fig. 1b). When exposed to just the black pepper treatment (100 %), the mean mortalities of the respective strains each exceeded 95 %; even the lowest treatment dose (10 %), with the exception of GAH at this dose, showed upwards of 70 % mean mortality. There were no significant differences in the mean mortalities induced in GAH versus those of either SILC or SUA (Table 3).

#### *Anopheles quadriannulatus*

The SANGWE strain was particularly susceptible to black pepper. Significantly higher mean mortalities were achieved in all pepper-inclusive treatments compared to the control, with approximately 90 % mortality observed even at the lowest pepper dose (ANOVA: *P* < 0.01; *F* = 145.06 at *df * = 17) (Fig. 1c).

#### *Anopheles funestus*

Other than the mortality of FANG exposed to the 100 % dose, which was less than in the 40 and 80 % doses, mortalities among *An. funestus* larvae generally increased with increasing proportions of black pepper. Generally, ingestion of black pepper did not cause more than 50 % mortality in either of the *An. funestus* strains. There was, however, significantly higher mortality among FANG larvae in the 40–100 % treatment doses than in the control (ANOVA: *P* < 0.01; *F* = 6.70 at *df * = 17) (Fig. 1d). For FUMOZ-R, although one-way ANOVA indicated a significant difference in mortality between the doses (*P* < 0.05; *F* = 3.51 at *df * = 17), none of the treatment mortalities differed significantly from the control. This is likely due to the wide variation in response to pepper ingestion (Fig. 1d). While this suggests that FUMOZ-R is less susceptible to black pepper than FANG, this was found to be statistically insignificant (Table 3).

### Piperine

**Fig. 3:**
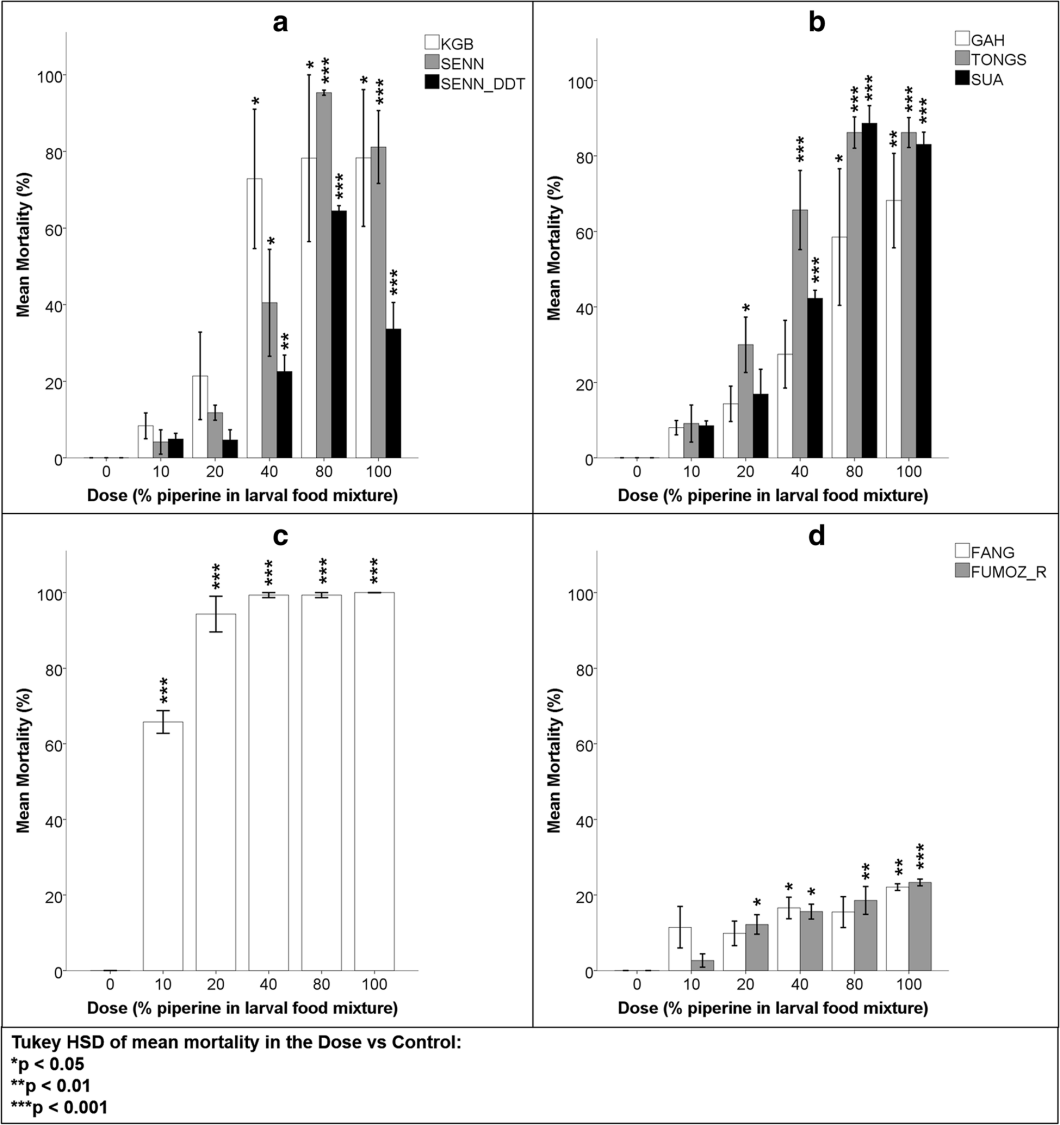
Mean mortalities of laboratory-reared *Anopheles* larvae 48 h after being fed powdered piperine. The dose value represents the percentage of piperine in a 50 mg treatment mixture. 0 % represents the control group which was fed standard larval food only and 100 % represents a treatment comprised of piperine only. A One-way ANOVA and Tukey HSD *post-hoc* comparisons were used for each strain to determine significant differences in mortality at each dose compared to that of the relevant control at 95 % confidence. **a** *Anopheles arabiensis* strains. **b** *Anopheles gambiae* (GAH& TONGS) and *An. coluzzii* (SUA) strains. **c** *Anopheles quadriannulatus.* **d** *Anopheles funestus* strains

Unlike black pepper, the powdered piperine did not spread effectively and if not scattered carefully over the surface area of water, it tended to clump. The larval clustering observed in the black pepper experiments did not occur in the piperine trials. All mortality results were recorded 48 h post-feeding with piperine (Fig. 3). The mortalities induced in SENN, SENN-DDT and GAH did not significantly differ from the mortalities in the *An. funestus* larvae, probably owing to high levels of variation around the means, whereas the mortalities in the other Gambiae complex strains were significantly higher than those recorded in the *An. funestus* strains (Table 4).

**Table 4:** Tukey HSD *post-hoc* analysis of *Anopheles* larval mean mortalities 48 h post-exposure to piperine

	Strain	Mean mortality	Standard deviation
1	KGB	51.83^7**,8–9*^	39.23
2	SENN	46.58^7***^	39.33
3	SENN-DDT	26.05^7***^	23.61
4	GAH	35.29^7***^	29.44
5	TONGS	55.43^7*,8–9**^	33.49
6	SUA	47.89^7**,8–9*^	34.65
7	SANGWE	91.75^1,6**,2–4,8–9***,5*^	14.11
8	FANG	15.09^1,6*,5**,7***^	6.91
9	FUMOZ-R	14.46^1,6*,5**,7***^	7.96

A one-way ANOVA (*P* < 0.01; *F* = 10.83 at *df * = 134), supplemented with a Tukey HSD *post-hoc* test, was used to determine significant differences in the overall mean mortalities induced between *Anopheles* strains indicated by superscript numbering at 95, 99 and 99.9 % confidence

*P < 0.05; **P < 0.01; ***P < 0.001

#### *Anopheles arabiensis*

All of the treatment mixtures including and exceeding 40 % piperine induced significantly higher mortality than the control in KGB (ANOVA: *P* < 0.01; *F* = 6.54 at *df * = 17), SENN (ANOVA: *P* < 0.01; *F* = 33.77 at *df * = 17) and SENN-DDT larvae (ANOVA: *P* < 0.01; *F* = 46.63 at *df * = 17) (Fig. 3a). However, the significantly higher mortalities achieved ranged from approximately 20–95 % among the strains; and while this was seemingly indicative of the strains having significantly different responses from each other, ANOVA showed otherwise for both KGB *versus* SENN-DDT and SENN *versus *SENN-DDT (Table 4).

#### *Anopheles gambiae* and *An. coluzzii*

In GAH, only the 80 and 100 % doses induced significantly higher larval mortality than the control (One-way ANOVA: *P* < 0.01; *F* = 7.95 at *df * = 17) and in both TONGS (ANOVA: *P* < 0.01; *F* = 40.05 at *df * = 17) and SUA (ANOVA: *P* < 0.01; *F* = 108.22 at *df * = 17), all dosages greater than and equal to 40 % piperine induced significantly higher mortalities than the control (Fig. 3b). There were no significant differences in mortality induced by piperine between either GAH and TONGS or GAH and SUA (Table 4).

#### *Anopheles quadriannulatus*

SANGWE larvae were more susceptible to the piperine than other *An. gambiae* complex strains (Table 4). Up to 100 % mortality was induced in larvae exposed to 100 % piperine and even 10 % piperine induced more than 60 % mortality. All of the piperine-inclusive doses induced significantly higher mortality than the control ( ANOVA: *P* < 0.01; *F* = 292.01 at *df * = 17) (Fig. 3c).

#### *Anopheles funestus*

FANG larvae only showed significantly higher mortalities than that of the control in the 40 and 100 % piperine dosages (ANOVA: *P* < 0.01; *F* = 5.14 at *df * = 17). However, the highest mean mortality, which was induced by 100 % piperine, was only 22 %. The mortalities induced by piperine in FUMOZ-R larvae were significantly higher than that of the control in all dosages exceeding 10 % piperine (ANOVA: *P* < 0.01; *F* = 17.88 at *df * = 17) although the highest mean mortality achieved was low at only 24 % using 100 % piperine (Fig. 3d). There was no significant difference in the mortality induced by piperine between FANG and FUMOZ-R (Table 4).

### Black pepper versus piperine

The SILC and TONGS strains were excluded from the statistical analyses comparing mortalities induced by black pepper versus piperine as neither were exposed to both. Mortality among the *An. gambiae* complex strains 24 h after being fed black pepper was significantly higher than that induced by piperine 48 h post-feeding (Student’s *t*-test: *P* < 0.01; *t *= 8.34 at *df * = 178). Although it appeared that black pepper induced higher mortality than piperine in the *An. funestus* strains as well, the difference is not statistically significant at 95 % confidence (Student’s *t*-test: *P* = 0.07; *t* = 1.87 at *df * = 58).

## Discussion

Prior to this study, *Anopheles* larval toxicity caused by the ingestion of either black pepper or piperine had not been explicitly investigated. To date, the evaluations of the insecticidal activity of *P. nigrum* has been focussed mainly on the constituent alkaloids and their effects on a variety of insect species ranging from those of economic importance [18, 19] to disease vectors such as *Aedes aegytpi*, *An. stephensi* and *Culex quinquefasciatus* [20, 21]. Of the compounds comprising *P. nigrum*, piperine is the most recognized and is abundant in fully differentiated shoots of the plant [22, 23].

Commercial, ground black pepper and piperine showed marked toxic effects against late instar larvae of all the *An. gambiae* complex strains tested. Piperine ingestion was evidently less toxic to *Anopheles* larvae than black pepper, but did elicit larval mortality over 48 h and may serve as a synergist amongst the various amides constituting black pepper. Black pepper and piperine induced mortality in insecticide susceptible and resistant *An. gambiae* complex strains with no statistically significant differences between them. This suggests that insecticide resistance mechanisms play no role in the detoxification of black pepper and piperine in these species.

The *An. funestus* strains, however, showed markedly lower sensitivities to black pepper and piperine. This may reflect differences in their feeding habits and metabolism. Alternatively, their larval rearing conditions, which partially reflect the characteristics of their natural breeding sites, may have influenced their relative sensitivities. This is because the FANG and FUMOZ-R *An. funestus* strains are reared in water containing algae obtained from a nearby fish pond whereas the *An. gambiae* complex larvae are reared in water containing no additives other than larval food. However, it is not clear as to how or why this may have affected their responses to black pepper and piperine. One possibility is that food sources in the algae reduced their propensity to feed on the pepper/piperine treatments, raising the question as to what effect competing multiple food sources would have on the efficacy of such treatments in natural breeding sites.

Larviciding is one of four types of larval source management strategies, the others being habitat modification, habitat manipulation and biological control. It is recommended mainly in areas where larval habitats are few, fixed and findable [6]. Where implemented, LSM serves as a useful supplementary vector control measure, targeting mosquitoes at a stage of their life-cycle where their mobility is limited to the body of water they inhabit. Currently employed larvicides must comply with stringent criteria established by the WHO Pesticide Evaluation Scheme (WHOPES). These include assessments of how hazardous they are to humans and the environment, their storage requirements and shelf life, the susceptibility of local vectors and the associated costs of utilizing them [6]. In terms of these standards, plant-derived products such as pepper and piperine may be particularly advantageous, depending on their efficacy and residual activity under field conditions. These data give no indication of the persistence of ground black pepper and piperine over time but it is envisaged that any pepper or piperine based control products would need to be formulated for increased persistence. Formulated products would also need to ensure adequate dispersal across a water surface, especially in the case of piperine which tended to clump.

A potential advantage of utilizing piperamides for larval control is that they act as neurotoxins, but in a manner distinct from pyrethroids, and so represent a novel mode of action [22], something urgently required for alternative methods of vector control [24]. They also have an inhibitory effect on enzymes and have shown synergistic effects when used in conjunction with pyrethrum [25]. They have low mammalian toxicity and are not environmentally persistent, degrading quickly under full sunlight [18, 26].

## Conclusions

*Piper nigrum* in the form of finely ground black pepper is highly toxic to members of the *An. gambiae* species complex including *An. gambiae* (*sensu stricto*), *An. coluzzii, An. arabiensis* and *An. quadriannulatus.* It appears markedly less toxic to *An. funestus.* Similarly, piperine, proved substantially more toxic to members of the *An. gambiae* species complex than to *An. funestus,* although piperine on the whole was less toxic than black pepper across all species and strains tested. There were no differences in response between insecticide-resistant and their corresponding insecticide-susceptible strains by species to intoxication by either black pepper or piperine, suggesting that insecticide resistance mechanisms have no effect on their relative susceptibilities to these compounds/derivatives. An appropriately formulated black pepper or derivative product may have potential as a larvicide for malaria vector control in settings where larval source management adds benefit under the auspices of integrated vector control. Assessments of the other piperamides present in black pepper may provide further insights into the larvicidal effects of this plant and its derivatives.

## References

[1] WHO (2014). World Malaria Report: 2014.

[2] Coetzee M, Hunt RH, Wilkerson R, della Torre A, Coulibaly MB, Besansky NJ (2013). *Anopheles coluzzii* and *Anopheles amharicus*, new members of the *Anopheles gambiae* complex. Zootaxa.

[3] Gillies MT, Coetzee M (1987). A supplement to the Anophelinae of Africa south of the Sahara.

[4] Sinka ME, Bangs MJ, Manguin S, Coetzee M, Mbogo CM, Hemingway J, Patil AP, Temperley WH, Gething PW, Kabaria CW, Okara RM, van Boeckel T, Godfray HCJ, Harbach RE, Hay SI. The dominant *Anopheles* vectors of human malaria in Africa, Europe and the Middle East: occurrence data, distribution maps and bionomic précis. Parasit Vectors. 2010;3:117. 10.1186/1756-3305-3-117 PMC3016360 21129198

[5] Knox TB, Juma EO, Ochomo EO, Pates Jamet H, Ndungo L, Chege P, Bayoh NM, N'Guessan R, Christian RN, Hunt RH, Coetzee M. An online tool for mapping insecticide resistance in major *Anopheles* vectors of human malaria parasites and review of resistance status for the Afrotropical region. Parasit Vectors. 2014;7:76. 10.1186/1756-3305-7-76 PMC3942210 24559061

[6] WHO (2012). Interim position statement: the role of larviciding for malaria control in sub-Saharan Africa.

[7] Killeen GF, Fillinger U, Knols BGJ (2002). Advantages of larval control for African malaria vectors: Low mobility and behavioural responsiveness of immature mosquito stages allow high effective coverage. Malar J.

[8] WHO (2013). Larval source management: a supplementary measure for malaria vector control: an operational manual.

[9] Fillinger U, Ndenga B, Githeko A, Lindsay SW (2009). Integrated malaria vector control with microbial larvicides and insecticide-treated nets in western Kenya: a controlled trial. Bull WHO.

[10] Maheu-Giroux M, Castro MC (2013). Impact of community-based larviciding on the prevalence of malaria infection in Dar es Salaam, Tanzania. PLoS ONE.

[11] Tehri K, Singh N (2015). The role of botanicals as green pesticides in integrated mosquito management – a review. Int J Mosq Res.

[12] Meghwal M, Goswami TK (2013). *Piper nigrum* and piperine: an update. Phytother Res.

[13] Bukhari T, Takken W, Koenraadt CJ (2011). Development of *Metarhizium anisopliae* and *Beauveria bassiana* formulations for control of malaria mosquito larvae. Parasit Vectors.

[14] Siddiqui BS, Gulzar T, Begum S (2002). Amides from the seeds of *Piper nigrum* Linn. and their insecticidal activity. Heterocycles.

[15] Gulzar T, Uddin N, Siddiqui BS, Naqvi SN, Begum S, Tariq RM (2013). New constituents from the dried fruit of *Piper nigrum* Linn., and their larvicidal potential against the dengue vector mosquito *Aedes aegypti*. Phytochem Lett.

[16] Hunt RH, Brooke BD, Pillay C, Koekemoer LL, Coetzee M (2005). Laboratory selection for and characteristics of pyrethroid resistance in the malaria vector *Anopheles funestus*. Med Vet Entomol.

[17] WHO (2005). Guidelines for laboratory and field testing of mosquito larvicides.

[18] Scott IM, Jensen H, Scott JG, Isman MB, Arnason JT, Philogène BJR (2003). Botanical insecticides for controlling agricultural pests: piperamides and the Colorado potato beetle *Leptinotarsa decemlineata* Say (Coleoptera: Chrysomelidae). Arch Insect Biochem.

[19] de Paula VF, de A Barbosa LC, Demuner AJ, Piló-Veloso D, Picanço MC (2000). Synthesis and insecticidal activity of new amide derivatives of piperine. Pest Manag Sci.

[20] Amer A, Mehlhorn H (2006). Larvicidal effects of various essential oils against *Aedes*, *Anopheles*, and *Culex* larvae (Diptera, Culicidae). Parasitol Res.

[21] Park IK, Lee SG, Shin SC, Park JD, Ahn YJ (2002). Larvicidal activity of isobutylamides identified in *Piper nigrum* fruits against three mosquito species. J Agric Food Chem.

[22] Scott IM, Jensen HR, Philogène BJ, Arnason JT (2008). A review of *Piper* spp. (Piperaceae) phytochemistry, insecticidal activity and mode of action. Phytochem Rev.

[23] Semler U, Gross GG (1998). Distribution of piperine in vegetative parts of *Piper nigrum*. Phytochemistry.

[24] Zaim M, Guillet P (2002). Alternative insecticides: an urgent need. Trends Parasitol.

[25] Jensen HR, Scott IM, Sims SR, Trudeau VL, Arnason JT (2006). The effect of a synergistic concentration of a *Piper nigrum* extract used in conjunction with pyrethrum upon gene expression in *Drosophila melanogaster*. Insect Mol Biol.

[26] Scott IM, Jensen H, Nicol R, Lesage L, Bradbury R, Sanchez-Vindas P, Poveda L, Arnason JT, Philogene BJR (2004). Efficacy of *Piper* (Piperaceae) extracts for control of common home and garden insect pests. J Econ Entomol.

[27] Oliver SV, Brooke BD (2014). The effect of multiple blood-feeding on the longevity and insecticide resistant phenotype in the major malaria vector *Anopheles arabiensis* (Diptera: Culicidae). Parasit Vectors.

[28] Nardini L, Christian RN, Coetzer N, Ranson H, Coetzee M, Koekemoer LL (2012). Detoxification enzymes associated with insecticide resistance in laboratory strains of *Anopheles arabiensis* of different geographic origin. Parasit Vectors.

[29] Kaiser ML, Koekemoer LL, Coetzee M, Hunt RH, Brooke BD (2010). Staggered larval time-to-hatch and insecticide resistance in the major malaria vector *Anopheles gambiae* S form. Malaria J.

[30] Amenya DA, Naguran R, Lo T-CM, Ranson H, Spillings BL, Wood OR, Brooke BD, Coetzee M, Koekemoer LL. Over expression of a cytochrome P450 (CYP6P9) in a major African malaria vector, *Anopheles funestus*, resistant to pyrethroids. Insect Molec Biol. 2008;17:19–25. 10.1111/j.1365-2583.2008.00776.x 18237281

[31] Wood OR, Hanrahan S, Coetzee M, Koekemoer LL, Brooke BD (2010). Cuticle thickening associated with pyrethroid resistance in the major malaria vector *Anopheles funestus*. Parasit Vectors.

